# Does IQ influence Associations between ADHD Symptoms and other Cognitive Functions in young Preschoolers?

**DOI:** 10.1186/1744-9081-10-16

**Published:** 2014-05-01

**Authors:** Nina Rohrer-Baumgartner, Pål Zeiner, Jens Egeland, Kristin Gustavson, Annette Holth Skogan, Ted Reichborn-Kjennerud, Heidi Aase

**Affiliations:** 1 Division of Mental Health, Norwegian Institute of Public Health, Oslo, Norway; 2 Oslo University Hospital, Child and Adolescent Mental Health Research Unit, Oslo, Norway; 3 Vestfold Hospital Trust, Tønsberg, Norway; 4 University of Oslo, Institute of Psychology, Oslo, Norway; 5 University of Oslo, Institute of Clinical Medicine, Oslo, Norway

**Keywords:** ADHD, IQ, Intellectual ability, Preschool, Cognition, Working memory, Language skills, Inhibition

## Abstract

**Background:**

Working memory, inhibition, and expressive language are often impaired in ADHD and many children with ADHD have lower IQ-scores than typically developing children. The aim of this study was to test whether IQ-score influences associations between ADHD symptoms and verbal and nonverbal working memory, inhibition, and expressive language, respectively, in a nonclinical sample of preschool children.

**Methods:**

In all, 1181 children recruited from the Norwegian Mother and Child Cohort Study were clinically assessed at the age of 36 to 46 months. IQ-score and working memory were assessed with subtasks from the Stanford Binet test battery, expressive language was reported by preschool teachers (Child Development Inventory), response inhibition was assessed with a subtask from the NEPSY test, and ADHD symptoms were assessed by parent interview (Preschool Age Psychiatric Assessment).

**Results:**

The results showed an interaction between ADHD symptoms and IQ-score on teacher-reported expressive language. In children with below median IQ-score, a larger number of ADHD symptoms were more likely to be accompanied by reports of lower expressive language skills, while the level of ADHD symptoms exerted a smaller effect on reported language skills in children with above median IQ-score. The associations between ADHD symptoms and working memory and response inhibition, respectively, were not influenced by IQ-score.

**Conclusions:**

Level of IQ-score affected the relation between ADHD symptoms and teacher-reported expressive language, whereas associations between ADHD symptoms and working memory and response inhibition, respectively, were significant and of similar sizes regardless of IQ-score. Thus, in preschoolers, working memory and response inhibition should be considered during an ADHD assessment regardless of IQ-score, while language skills of young children are especially important to consider when IQ-scores are average or low.

## Background

Attention Deficit/Hyperactivity Disorder (ADHD) is a neurodevelopmental disorder with a typical onset in preschool years. This period is important for cognitive development. For instance, elementary forms of Executive Functions (EF) components, such as working memory (WM) and inhibition, are already emerging before the age of three and develop rapidly in preschool years [1]. Language also develops at a rapid pace in this period and is often characterized by a transition from two word utterances in toddlerhood to complex sentences in late preschool years [2]. Children with high levels of ADHD symptoms often show delays or impairments in cognitive functions such as WM, inhibition, and language [3-9]. These delays are often present from an early age and can have a substantial impact on children’s academic and social functioning [10-12]. However, our understanding of associations between ADHD symptoms and cognitive functions in preschoolers is limited and mainly based on parent reports. Teacher reports of language skills would be particularly useful as the informants usually have a large “reference base” for comparison, from years of experience with children of the same age, representing the full range of language skills. Teacher reports provide information about language skills that may be challenging to elicit in a test situation with young preschoolers due to shyness and/or limited motivation or attention [13] and they are easy and inexpensive to administer.

Children with ADHD have been shown to have lower IQ-scores [14]. The term “IQ-score” refers to the performance on a limited set of standardized, cognitive tests which were originally combined to measure mental age, whereas “cognitive functions” is a broader term and refers to several specific cognitive functions, such as memory and language skills. Intellectual ability contributes to mental health already in young childhood. Four year old children with below average IQ-scores have been found more likely to experience mental health problems at school age [15]. Recent studies further indicate that associations between ADHD and cognitive functions depend on IQ and state that IQ-score therefore should be taken into account when investigating cognitive functions in ADHD [16,17].

A number of studies have reported inconsistent findings regarding deficits in WM and inhibition in preschoolers with ADHD [18]. For example, weaker associations between cognitive functions and ADHD symptoms have been found in preschoolers than those typically found in older children [19] or no interaction between ADHD symptoms and IQ-score on either verbal or nonverbal WM have been reported [20].

In older children, a strengthening of associations between inattention, verbal WM, and verbal memory has been shown to occur through the school years [21,22]. Contrary to this, it has also been suggested that children with ADHD have a developmental delay of verbal WM only until eight years of age [23]. It is unclear why these results are showing a contrary age-effect regarding verbal WM compared to previous studies, however, differences in cognitive measures and in sample characteristics might play a role.

Combined, findings from most preschool- and school aged samples raise the question if associations between ADHD symptoms and WM are strengthened with age. Cognitive functions must have reached a certain level of maturity before delays or deficits are visible and executive aspects of WM are just emerging at the age of three [24,25]. Based on previous research and a theoretical assumption, certain hypotheses about the influence of IQ-score on the association between ADHD symptoms and cognitive functions in preschoolers can be made. Since stronger associations between ADHD symptoms and verbal memory have been found with increasing age in several studies and since young children with higher IQ-scores may have equivalent cognitive functioning as slightly older children with average intellectual development, it is possible that an association between ADHD symptoms and verbal WM grows stronger with increasing *mental* age/IQ-score. Hence, IQ-score could moderate the association between ADHD symptoms and nonverbal WM. Put differently, an interaction between IQ-score and ADHD symptoms on verbal WM might be expected: the higher the IQ-score, the stronger the association between ADHD symptoms and verbal WM. On the other hand, according to Sowerby and colleagues [23], weaker associations between ADHD symptoms and verbal WM with increasing IQ-score could be expected. There is also another reason for expecting a decrease in associations. As verbal WM is closely associated with language [26,27], which in turn is closely associated with IQ-score as verbal ability is an important aspect of intellectual ability, verbal WM may be well-developed in children with high IQ-scores regardless of ADHD symptoms. In fact, weaker associations between ADHD symptoms and verbal WM with increasing IQ-score can also be expected with regard to nonverbal WM, as nonverbal WM is assumed to be language based as well [28]. However, not all studies have found support for age-related associations between ADHD and nonverbal WM [29]. In the light of these conflicting findings, in which methodological differences may explain some of the inconsistency, we leave predictions about a possible interaction between IQ and ADHD symptoms on WM open.

An interaction between ADHD symptoms and IQ-score on response inhibition is not likely, as impaired inhibition is closely related to ADHD symptoms [4,5], and considerably less so with IQ-score [30]. ADHD symptoms may therefore be associated with impaired response inhibition regardless of IQ-score. On the other hand, inhibition has been found to be more strongly associated with ADHD symptoms in young school-aged children than in older, which would imply decreasing associations between ADHD symptoms and inhibition with age [22], however, we do not know whether this process is linear and whether it already starts in preschool years.

We would expect weaker associations between ADHD symptoms and preschool teacher-reported language skills with higher IQ-score, for the same reason as expecting weaker associations between ADHD symptoms and verbal WM with increasing IQ-score. As teacher-reported expressive language skills are closely related to intellectual ability [31], high IQ-score may be associated with well-developed expressive language skills regardless of ADHD symptoms in the present study, while children with lower IQ-scores may struggle with language, making an interaction between ADHD symptoms and IQ-score on preschool teacher-reported expressive language skills likely.

In sum, a higher IQ-score may either make cognitive problems related to ADHD symptoms more visible or it may act as a protector from the impact of such cognitive deficits, or there may be no effect of a high IQ-score on the association between ADHD symptoms and cognitive functions in preschoolers. In order to investigate these possible interactions, nonclinical samples should be used, minimizing the risk of studying samples with disproportionally few children with well-developed cognitive functioning and/or higher IQ-scores. The use of nonclinical samples may be particularly important when studying preschoolers, as the classification of ADHD in young children is challenging. Yet to our knowledge, there are no studies with nonclinical samples of preschoolers to date specifically investigating the impact of IQ-score on the association between ADHD symptoms and cognitive functions.

The main aim of this study was to test whether IQ-score influences associations between ADHD symptoms and WM, response inhibition, and reported expressive language, respectively, using a nonclinical sample of preschoolers. In order to address the main aim, we first investigated if there were significant associations between ADHD symptoms and the selected cognitive functions and in what direction they went and then went on to test whether there were significant interactions between ADHD symptoms and IQ on the cognitive functions mentioned above. A significant interaction would mean that the association between ADHD symptoms and the cognitive function under study depended on the level of IQ-score. To increase the clinical value of the results, follow-up analyses investigated whether negative associations between ADHD symptoms and selected cognitive functions differed in children with below median IQ-score compared with children with above median IQ-score.

## Methods

The present study is a sub study of a longitudinal study of ADHD in preschool children. The longitudinal study recruited its participants from the Norwegian Mother and Child Cohort Study (MoBa) [32], a population based birth cohort at the Norwegian Institute of Public Health. The parents of the participating children gave informed consent to participate in the research and to the publication of the results. The present study was approved by the Norwegian Regional Ethics Committee for Medical and Health related Research.

### Participants

**Figure 1:**
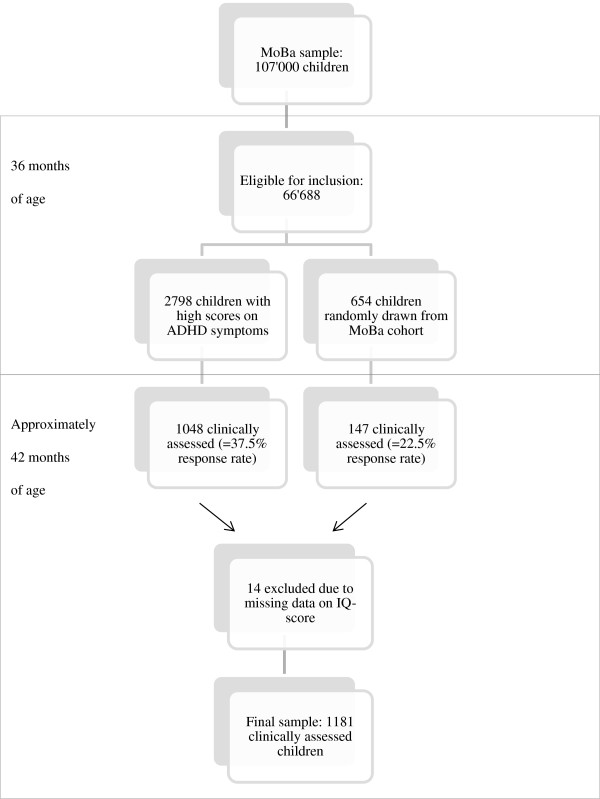
**Flowchart of recruitment. ***Note*: MoBa = Norwegian Mother and Child Cohort Study. Eligible for inclusion = children born between April 2004 and January 2008. Of the 66’688 children eligible for inclusion, 149 children were excluded due to severe medical conditions and/or high levels of autism symptoms.

The present study used a nonclinical sample of young preschool children recruited from the MoBa. A flowchart of the recruitment of the sample is presented in Figure 1.

In the MoBa, all parents received a questionnaire when their child was 36 months old. The questionnaire included 11 questions regarding hyperactivity, impulsivity and attention problems from the Child Behavior Checklist (six questions) [33] and diagnostic criteria for ADHD (five questions) [34], that were used to select participants. In order to oversample children with relevant symptoms, about 80% of invited children were those who scored at or above the 90^th^ percentile on these questions. Children were also invited to participate when hyperactivity was reported as a health problem. Children with severe medical conditions compromising their ability to conduct the clinical assessment and/or high levels of autistic symptoms were excluded from the study before invitations were sent (n = 149). A total of 2798 children were invited from the MoBa cohort during the period from August 2007 to January 2011on the basis of these criteria. Of these, 1048 children (37.5%) participated in the clinical assessments. An additional 654 randomly drawn children from the MoBa cohort were invited, of whom 147 (22.5%) participated. The total sample of children who were clinically assessed at the mean age of 42 months was therefore 1195. Of these, the present study included the 1181 children who had valid IQ-scores (618 boys and 563 girls), all aged between 37 to 46 months. Additional information was provided by parents and preschool teachers (see below).

### Procedure

Prior to clinical investigation, a questionnaire about expressive language (see below) was sent to the preschool teachers of all the participating children. The one-day assessment included neuropsychological tests and a semi-structured interview with a parent. The neuropsychological assessment lasted for approximately 1.5 hours including breaks, and small incentives (stickers, small cookies, grapes or raisins) were at random time points given to the child to improve motivation during the neuropsychological assessment. Each child was individually evaluated by a psychologist while one parent was present. The interviews were conducted by psychologists, psychiatrists, or by trained psychology students. The examiners did not know if the child had been selected due to high levels of ADHD symptoms or if the child had been randomly drawn from the MoBa cohort. None of the children in the sample received psychopharmacological treatment at the time of the assessment.

### Measures

The present study included measures covering selected cognitive functions associated with ADHD symptoms.

#### DSM-IV criteria for ADHD

For assessment of symptoms of DSM-IV disorders, including ADHD, we used the Preschool Age Psychiatric Assessment (PAPA) [35]. The PAPA is a semi-structured, clinical interview conducted with a parent. It provides information about the scale and frequency of symptoms, and about impairment, according to diagnoses in DSM-IV. In the present study we used this interview to assess the number of inattention-, hyperactivity-, and impulsivity symptoms, which add up to the total amount of ADHD symptoms, and symptoms of oppositional-defiant disorder (ODD). The PAPA interview rates ADHD symptoms on an unidimensional scale equivalent to the ADHD-Rating Scale IV [36], which is often used with older children. Impairment was recorded present when parents reported that the child experienced significant impairment due to his/her ADHD symptoms in at least one area of functioning.

The PAPA is the only comprehensive psychiatric interview to our knowledge with demonstrated test-retest reliability and validity for assessing psychiatric symptoms and disorders in toddlers and preschool children. We assessed interrater reliability of the number of DSM-IV symptoms and impairment scores by rescoring tapes of 79 randomly chosen PAPA interviews by raters blind to knowledge about the child. The average intraclass correlations (ICC) were .99 for number of inattention symptoms, .97 for hyperactivity symptoms, .96 for impulsivity symptoms, and .94 for total impairment score.

#### IQ-score, verbal intellectual ability, and nonverbal intellectual ability

The Stanford-Binet 5^th^ revision (SB-5) [37] is a standardized test battery with good psychometric properties [38] that is widely used. The stop rule of discontinuing the test after four consecutive null scores was applied in all tests from this battery.

IQ-score was estimated from a short version of the Stanford-Binet 5^th^ revision; the “vocabulary” task was used to assess verbal intellectual ability and the “object matrices” task was used to measure nonverbal intellectual ability. Together, these two measures provide an estimate of abbreviated IQ.

#### Working memory

The tasks used for measuring WM in the present study were selected based on the assumption that these tests require preschoolers to rehearse or manipulate information while either listening to the rest of the information presented in the test item or while waiting to respond. The subtask “memory for sentences” from the Stanford-Binet 5^th^ revision was included as a measure of verbal WM. In this task, the child is asked to repeat sentences of increasing length.

Nonverbal WM was measured with the subtasks “delayed response” and “block span”. The first subtask consists of three cups under one of which a toy is hidden. The cups are either concealed for a few seconds before the child points to the correct cup (delayed response paradigm) or the cups switch places after the toy has been hidden. The “block span” subtask is a visual span task where the child has to tap blocks in the same order as demonstrated by the test administrator. Scaled scores from both WM tasks were used.

#### Inhibition

The “Statue” task from the Developmental Neuropsychological Assessment (NEPSY) [39], which is standardized for 3–12 years old children, was used to assess response inhibition. During the task, the children were asked to stand still (“like a statue”) with eyes closed and to remain silent for 75 seconds, while the test administer produced several timed, distracting stimuli. For every 5-second interval without utterances or significant movements two points were awarded, and one point was awarded for every 5-second interval with one utterance or movement. More than one movement or utterance resulted in zero points for that interval. Raw scores were used. The highest possible raw score was 30. Smiling, slight finger movements and involuntary coughing were allowed. Reminders of standing still and being silent could be given after utterances and movements.

The NEPSY was chosen for this study because it is a well-known neuropsychological test battery for preschoolers with subtests of short duration. The tasks are appealing for young children, which is important when assessing cognitive functions of 3-year olds. The NEPSY is also one of few test batteries for preschoolers that have been translated to Norwegian. Regarding psychometric properties of the “Statue task”, only test-retest reliability from the American manual are reported, which is limited (.50) [40]^a^.

#### Expressive language

The Child Development Inventory (CDI) [41], whose subscale measuring expressive language was completed by preschool teachers in the present study, is a questionnaire for assessing children aged 15 months to 6 years. The subscale used consists of 50 items assessing mainly expressive communication, from simple gestural (one item: “Points to things”), vocal (three items about pronunciation) to complex language expression. Each item was scored yes (1) or no (0) according to the teacher’s view of the child’s skills, resulting in a possible maximum raw score of 50. The CDI has been found to have good sensitivity and specificity for identifying preschool children with delayed development, and CDI scores have been found to be consistent with children’s results on psychometric tests [42].

### Data management and statistical analyses

Despite their young age, the majority of children completed all the tests. Participants were only included in the study if they had less than six missing items (less than 10% missing) on the CDI questionnaire from teachers. SPSS version 20 was used to analyse the data. Number of ODD symptoms and parental education in years were considered as potential confounders and are therefore presented in Table 1. They were not included in the final analyses for the following reasons: controlling for parental education when studying cognitive functions related to ADHD symptoms reduces the variance of interest, thereby minimizing chances of finding relevant associations and reducing external validity. Also, the inclusion of parental education in the analyses did not lead to significant changes in the results. The number of ODD symptoms was not significantly associated with any of the cognitive measures or with expressive language and a recent study showed that ADHD symptoms and their associated cognitive problems, not co-morbid ODD, are related to academic impairment [10].

**Table 1:** Participant characteristics and descriptive statistics

**Variable**	***Min***	***Max***		***M***	***SD***
Gender (% boys/girls) (N = 1181)			52.3/47.7		
Age in years *(N = 1181)*	3.1	3.9		3.5	0.1
Mothers’ edu. (in years) (N = 1132)	9	18		15.2	2.4
Fathers’ edu. (in years) (N = 1087)	9	18		14.5	2.6
Mothers’ age (N = 1180)	19	43		30.6	4.3
ADHD symptoms (N = 1181)	0	18		4.0	3.9
ODD symptoms (N = 1181)	0	8		1.4	1.5
IQ (N = 1181)	67	130		101.9	9.2
Verbal ability (N = 1181)	2	17		9.7	2.1
Nonverbal ability (N = 1181)	4	18		10.9	2.0
VWM (N = 1125)	2	16		10.8	2.9
NVWM (N = 1174)	2	17		10.4	2.8
Response inhibition (N = 1031)	0	30		14.7	8.4
Reported expr. language (N = 1067)	8	50		41.6	7.3

*Note.* Edu. = education. Mothers’ age = age in years at the time of childbirth. Symptoms = number of symptoms. IQ = abbreviated IQ score estimated from the verbal ability and nonverbal ability subtasks from the Stanford-Binet-5 (SB-5). VWM = verbal working memory from SB-5. NVWM = nonverbal working memory from SB-5. The SB-5 subtask scores were standardized, with Mean = 10 and SD = 3. Response inhibition = statue task (NEPSY), raw scores. Reported expr. language = reported expressive language = CDI from preschool teachers, raw scores.

Before investigating the main aim of the study, we checked that each association between ADHD symptoms and verbal and nonverbal WM, expressive language, and response inhibition was significant when gender and IQ-score were controlled for (Table 2). This was done with four multiple regression analyses with the three cognitive measures and expressive language skills, respectively, as dependent variables. Assumptions for multiple regression analyses were met except for a slight risk of heteroscedasticity (judging from the scatter plot in the SPSS output) in the analysis where teacher-reported expressive language was the dependent variable. Therefore, this analysis was rerun with Generalized Linear Models, using the robust covariance matrix in order to obtain standard errors that were robust to violation of the assumption of homoscedasticity (results not shown). As the results did not differ notably from the original linear regression analysis, the initial analysis was kept.

**Table 2:** Regression analyses summary for ADHD symptoms predicting cognitive measures and reported expressive language skills

**Measure**	**Variable**	***B***	***SE B***	**β**	***t***	***p***	***R***^***2***^
**VWM**							
	ADHD symptoms	-0.07	0.02	-.09	-3.26	.001	.02
	IQ	0.10	0.01	.30	10.52	<.001	
	Gender	0.14	0.17	.02	0.83	.406	
**NVWM**							
	ADHD symptoms	-0.10	0.02	-.14	-5.04	<.001	.03
	IQ	0.05	0.01	.18	6.18	<.001	
	Gender	0.42	0.16	.08	2.66	.008	
**Response inhibition**							
	ADHD symptoms	-0.35	0.07	-.16	-5.21	<.001	.03
	IQ	0.08	0.03	.09	2.90	.004	
	Gender	2.81	0.51	.17	5.48	<.001	
**Reported expressive language**							
	ADHD symptoms	-0.26	0.06	-.14	-4.65	<.001	.03
	IQ	0.20	0.02	.25	8.48	<.001	
	Gender	1.41	0.43	.10	3.30	.001	

*Note.* Analyses were controlled for IQ and gender. ADHD symptoms = number of symptoms. IQ = abbreviated IQ-score estimated from the verbal ability and nonverbal ability subtasks from the Stanford-Binet-5 (SB-5). VWM = verbal working memory (SB-5). NVWM = nonverbal working memory (SB-5). Reported expressive language = preschool teacher-reported expressive language (CDI). R^2^ reported for ADHD symptoms predicting cognitive measures. Gender: significant, positive betas mean that girls outperformed boys.

In the main analyses, multiple regression analyses were used to study possible interactions between IQ-score and ADHD symptoms on verbal WM, nonverbal WM, response inhibition, and expressive language while controlling for gender (Table 3). The independent variables IQ-score and number of ADHD symptoms were centred before their product was computed. The interaction effects show the degree to which associations between ADHD symptoms and the cognitive measures differed depending on IQ-score.

**Table 3:** Interactions between ADHD symptoms and IQ on cognitive measures and reported expressive language skills

**Measure**	**Variable**	**B**	***SE B***	**β**	***t***	***p***	***R***^***2***^
**VWM**	ADHD symptoms *IQ	0.00	0.00	-.02	-0.84	.400	.11
**NVWM**	ADHD symptoms *IQ	0.00	0.00	.00	0.10	.920	.07
**Response inhibition**	ADHD symptoms *IQ	0.00	0.01	.00	0.12	.903	.07
**Reported expressive language**	ADHD symptoms *IQ	0.01	0.01	.06	2.14	.032	.11

*Note.* ADHD symptoms = number of symptoms. IQ = abbreviated IQ-score estimated from the verbal ability and nonverbal ability subtasks from the Stanford-Binet-5 (SB-5). VWM = verbal working memory (SB-5). NVWM = nonverbal working memory (SB-5). Reported expressive language = preschool teacher-reportedexpressive language (CDI). The analyses also included ADHD symptoms, IQ, and gender.

In addition to running the interaction analyses with multiple regression, we chose to split the sample in two at the median IQ-score (103) to provide an illustration of the differences in the associations, or lack thereof, at different levels of intellectual ability. The median was chosen as it is close to the average IQ-score and as it created two groups of similar sizes. The sample consisted of 562 children (325 boys, 237 girls) with below median IQ-score and 619 children (293 boys, 326 girls) with median or above median IQ-score. Multiple regression analyses were used to study associations between ADHD symptoms and the four separate cognitive measures in the “median and above median group” and in the “below median group” while controlling for gender.

## Results

Demographic characteristics and descriptive data are presented in Table 1.

The sample was very homogeneous regarding age and the children were mostly from families with highly educated parents. We estimated the annual income of the MoBa, from which the sample was drawn, to equal the mean income in Norway. The sample of the present study had more ADHD symptoms and ODD symptoms than one would expect to find in the normal population. The means and standard deviations of the cognitive measures of the whole sample were approximately as one would expect to find in the normal population.

Table 2 shows associations between ADHD symptoms, cognitive measures, and teacher-reported expressive language skills, controlled for IQ-score and gender.

Both IQ-score and ADHD symptoms were significantly associated with WM, response inhibition, and reported language skills. Girls outperformed boys on the measures of NVWM, response inhibition and on reported expressive language, while there were no gender differences on the VWM measure.

Table 3 shows interaction effects between ADHD symptoms and IQ-score on cognitive measures and teacher-reported expressive language skills.

There was a significant interaction effect between ADHD symptoms and IQ-score on teacher-reported expressive language skills, not on WM or response inhibition.

**Figure 2:**
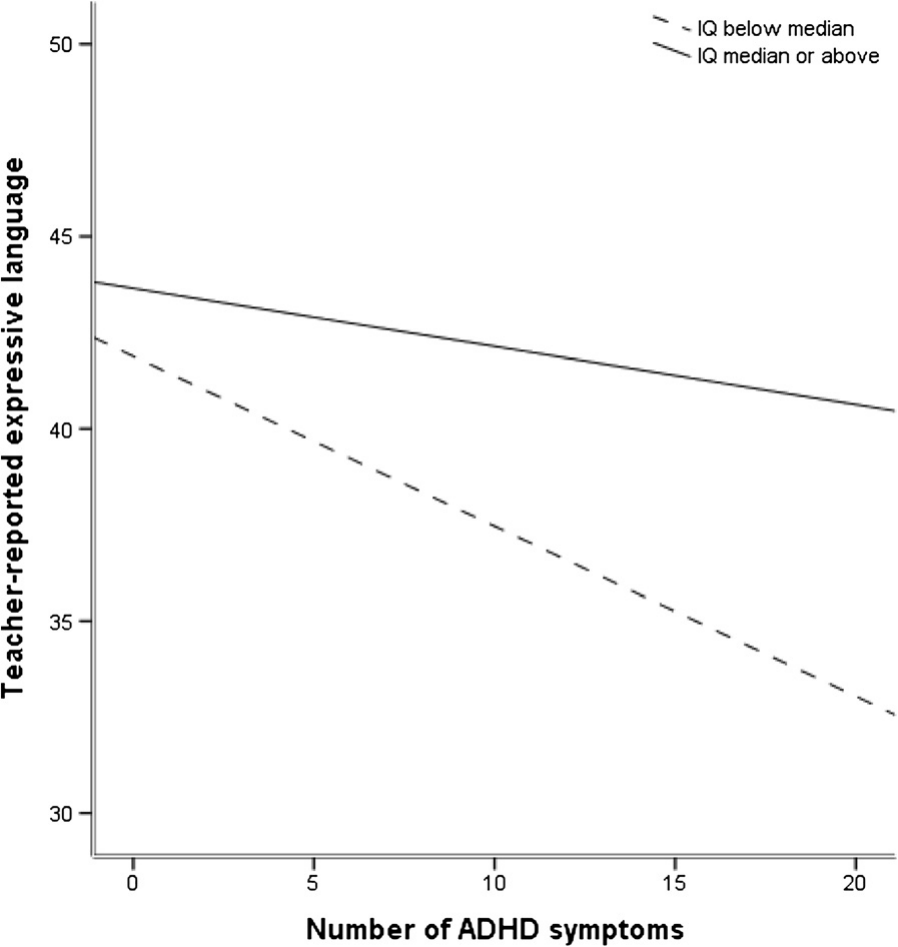
**Associations between ADHD symptoms and expressive language in the below-median and in the median-and-above-median-IQ group.***Note.* Teacher-reported expressive language = CDI from preschool teachers, raw scores. ADHD symptoms were assessed with the PAPA.

Figure 2 is a visual presentation of the significant interaction effect in Table 3. It illustrates how the association between ADHD symptoms and teacher-reported expressive language skills differed depending on intellectual ability level. According to Cohen, Cohen, West, and Aiken [43], plotting an interaction is the first step to its interpretation.

Furthermore, follow-up analyses were performed to test if the findings of no interaction between ADHD symptoms and IQ-score on either VWM, NVWM, or response inhibition were due to limited statistical power. These regression analyses controlled for gender showed that associations between ADHD symptoms and verbal WM and nonverbal WM, respectively, were of similar sizes in both IQ-divided groups (verbal WM: below median IQ group (n = 525): B = -0.07, SE B = 0.03, β = -.09, t = -2.09, p = .037, R^2^ = .1; median or above median IQ group (n = 600): B = -0.10, SE B = 0.03, β = -.14, t = -3.48, p = .001, R^2^ = .2; nonverbal WM: below median IQ group (n = 558): B = -0.13, SE B = 0.03, β = -.18, t = -4.43, p < .001, R^2^ = .5; median or above median IQ group (n = 616): B = -0.09, SE B = 0.03, β = -.13, t = -3.15, p = .002, R^2^ = .2). Associations between ADHD symptoms and response inhibition were also of similar sizes in both groups: below median IQ group (n = 482): B = -0.36, SE B = 0.10, β = -.17, t = -3.80, p < .001, R^2^ = .07; median or above median IQ group (n = 549): B = -0.37, SE B = 0.10, β = -.16, t = -3.85, p < .001, R^2^ = .05. However, associations between ADHD symptoms and reported expressive language skills were not similar in size in the two groups (below median IQ group (n = 507): B = -0.42, SE B = 0.09, β = -.21, t = -4.95, p < .001, R^2^ = .07; median or above median IQ group (n = 560): B = -0.14, SE B = 0.07, β = -.08, t = -2.00, p = .046 R^2^ = .01).

## Discussion

IQ-score and ADHD symptoms were significantly associated with WM, response inhibition, and teacher-reported language skills in our sample of children. Girls outperformed boys on most measures. ADHD symptoms had a differential effect on teacher-reported expressive language depending on IQ-score: in children with below median IQ-score, a larger number of ADHD symptoms were more likely to be accompanied by poorer teacher scores of expressive language skills, while the level of ADHD symptoms exerted a smaller effect on reported language skills in children with above median IQ-score. As for the other cognitive measures, no such interactions were found, as associations between ADHD symptoms and WM and response inhibition, respectively, were significant and of similar sizes regardless of IQ-score. Thus, our hypotheses regarding teacher reports of expressive language and response inhibition were supported.

We found no interaction between IQ-score and ADHD symptoms on either verbal WM or nonverbal WM. This could mean that the association between ADHD symptoms and WM is strengthened with increasing IQ-score in some children, while the association is weakened with increasing IQ-score in others. This interpretation would partially support previous studies [21-23], if the assumption that chronological and mental age show similar patterns is correct. We did not find support for the assumption that WM is well-developed in children with high IQ-scores regardless of ADHD symptoms, due to its close association with language [26,27]. Instead, the results indicate that young preschoolers with high levels of ADHD symptoms can struggle with reduced WM regardless of IQ-score. The same conclusion was reached for response inhibition, as it was associated with ADHD symptoms regardless of IQ-score. Inhibition is assumed to be one of the first executive functions to develop [44], making inhibitory deficits a central feature of ADHD already at a young age [45].

Some aspects of the design of the present study must be discussed as IQ-score is interrelated with ADHD symptoms and with other cognitive functions. However, previous studies have shown that unique aspects of cognitive functions contribute to IQ-score [46], and that impaired executive functions, e.g. response inhibition and WM, do not account for lower IQ-scores in children with ADHD [47,48]. Expressive language skills, on the other hand, are likely to be more closely connected with IQ-score. This may be particularly true for teacher-reports of language [31]. The finding of an interaction between intellectual functioning and ADHD symptoms on teacher-reported expressive language in the present study supports this finding [31]. Teacher-reported expressive language was included in the present study as it was measured independently from the other constructs under study (ADHD symptoms were reported by parents and WM and response inhibition were assessed with neuropsychological tests). It is also a convenient measure of language and could thereby facilitate more frequent considerations of language skills in ADHD assessments. Teacher reports of expressive language skills may be particularly useful when assessing young preschoolers as many of these tend to be shy when required to speak in direct assessments, whereas receptive language (which usually includes pointing at pictures in response to a question) is more easily assessed in this age group.

### Strengths and limitations of the present study

Strengths of the present study include a large sample with a nearly even distribution of boys and girls, an assessment with a well-validated interview for preschoolers (PAPA) to assess psychiatric symptoms, dimensional characterization of ADHD symptoms, and the use of performance-based measures of the key executive functions WM and response inhibition that are developmentally appropriate for 3-year olds. Moreover, the present sample is drawn from a nonclinical cohort, increasing the likelihood of having a sample with normally distributed IQ-scores. In contrast, clinical samples usually include more severe cases of ADHD, favouring children with low IQ-scores. On the other hand, there is the risk of underrepresentation of these children in the present study, which could constitute a limitation. The initial participation rate of the MoBa was 38.7% [49] and additional attrition occurred at later stages. By 36 months of age, when the children were invited to join the present study, response rate was down to 58.5% [49]. The clinical part of the study had a participation rate of 35% (unpublished). Thus, there is a risk of a self-selection bias i.e. that the participants differ systematically from nonparticipants. However, there were no statistically significant differences between clinically assessed children and invited children who did not participate in the clinical part of the study or between participants in the present study and the whole MoBa cohort with regard to background demographic characteristics and pre- and perinatal risk factors. The only exception was a slightly higher level of maternal education in assessed children in the present study (unpublished). Therefore, the sample in the clinical study is very similar to the sample in the whole MoBa. The risk of a selection bias in the MoBa and in a sub study of autism in the MoBa has been thoroughly discussed by Nilsen and colleagues [50,51]. Children participating in the MoBa are less likely to come from single-parent households, have young mothers (below the age of 25), and to have mothers who smoke [50], which is similar to the selection bias found in the Danish birth cohort [52]. Although prevalence rates in the MoBa cannot be assumed to be representative of the general population, associations between variables, which is the focus of the present study, do not seem to be affected [50,51]. However, the Danish study shows that although the majority of associations studied are not affected by selection bias, some can be [52]. We can therefore not exclude this possibility in our study. Children with higher IQ and well-developed language skills could be overrepresented in the present study due to the higher levels of maternal education, as these traits are correlated and highly heritable. However, this is unlikely to be a concern as the present study does not have a restricted range of IQ, as the minimum IQ was 67 and the maximum IQ was 130. Other possible limitations in the study include the use of an abbreviated IQ instead of a full scale IQ. The abbreviated IQ was chosen due to the young age of the children which only allows a relatively brief direct assessment. Furthermore, power estimates showed that the present study did not have enough statistical power for significance testing of the categorical follow-up analyses. We acknowledge the possibility that the selection of different tests and measures could have resulted in different results. This is a general limitation in neuropsychological research; however, it is particularly relevant for the statue task from the NEPSY battery that was used in the present study, as this task has limited psychometric properties. Future studies will have to clarify if the present results are task-related.

## Conclusions

IQ-score was found to influence the association between preschool teacher-reported expressive language skills and ADHD symptoms. In young preschool children with higher IQ-scores, ADHD symptoms were more weakly associated with delayed expressive language than in children with lower IQ-scores. IQ-score did not influence associations between ADHD symptoms and WM and response inhibition, respectively.

Findings of the present study show that language skills of children should be considered when an ADHD assessment is undertaken, especially when intellectual ability is average or low. This is not routinely done, even though language delay can have considerable negative consequences at school age [53]. Furthermore, our findings indicate that preschool teachers and clinicians need to be aware that children with higher intellectual ability can still suffer from significant ADHD symptoms and related cognitive difficulties. The clinical implications of the present study should be viewed in the context of previous studies which have shown that neuropsychological assessments and early interventions are important for better prognosis for children with ADHD [54] and that the enhancement of neuropsychological functioning in preschool years holds promise for improving prognosis for children with ADHD [55]. Delays or impairments in WM, response inhibition, and expressive language affect communication and social interaction, both of which are related to prognosis [56].

Future studies are encouraged to consider the intellectual ability of their sample before generalizing findings about ADHD symptoms and teacher-reported language skills. The results of the present study suggest that associations between ADHD symptoms and teacher-reported language skills are more likely to occur in samples with lower IQ-scores than in samples with well-developed intellectual ability.

## Endnote

^a^Corrections issued from the Publisher of the NEPSY test (Psykologiförlaget AB), 2002, were used.

## Abbreviations

WM: Working memory.

## Competing interests

None of the authors have competing interests.

## Authors’ contributions

NR-B contributed to the clinical assessment of the participants, study design, literature searches, statistical analyses, interpretation of results, and writing of the manuscript. PZ and HA are scientifically and financially responsible for the design of the Norwegian Longitudinal ADHD Study (from which the data of the present study were drawn), including selection of measures and recruitment of participants for the present study, they contributed to the interpretation of results and to revising the manuscript critically for intellectual content. JE contributed to the design of the study, the interpretation of results and to revising the manuscript critically for intellectual content. KG contributed to the design of the study, statistical analyses, interpretation of results, and to revising the manuscript critically for intellectual content. AHS contributed to the clinical assessment of the participants, took part in the discussion of neuropsychological concepts under study and contributed to revising the manuscript critically for intellectual content. TR-K contributed to the design of the Norwegian Longitudinal ADHD study and the present study, interpretation of results and to revising the manuscript critically for intellectual content. All authors read and approved the final manuscript.
